# A Comprehensive Review of the Mechanisms of Human Q Fever: Pathogenesis and Pathophysiology

**DOI:** 10.3390/pathogens14060589

**Published:** 2025-06-14

**Authors:** José-Luis Pérez-Arellano, Jose Curbelo, Cristina Carranza-Rodriguez

**Affiliations:** 1 University Institute of Biomedical and Health Research (Instituto Universitario de Investigaciones Biomédicas y Sani tarias IUIBS), University of Las Palmas de Gran Canaria (ULPGC), 35016 Las Palmas, Spain; cristinacarranzarodriguez@gmail.com; 2 Department of Medicine, University Francisco de Vitoria, 28223 Madrid, Spain; curbelo1984@gmail.com

**Keywords:** *Coxiella burnetii*, pathogenesis, pathophysiology

## Abstract

*Coxiella burnetii* infection has a worldwide distribution, although the incidence and clinical manifestations vary between and within countries. There are the following four basic forms: asymptomatic infection, acute Q fever, chronic Q fever, and post-Q fever fatigue syndrome. The aim of this review is to provide a comprehensive overview of the important aspects of its pathogenesis and pathophysiology. First, we provide a brief update of the taxonomic aspects, basic structures, and genotypes of *C. burnetii* necessary for the proper interpretation of the following sections. Routes of infection, different stages of pathogenesis (respiratory entry of *C. burnetii*; penetration into alveolar macrophages, life cycle, and effects; systemic dissemination), and innate, acquired humoral and cell-mediated immune responses in different forms of infection are described in detail. The pathophysiology and clinical manifestations of Q fever, such as the main mechanisms of injury, in isolation and in combination, are reviewed. The clinical and biological manifestations of the two main forms of Q fever (acute and chronic) are outlined, with a brief definition and mention of the mechanisms of post-Q fever fatigue syndrome.

## 1. Introduction

Q fever is an anthropozoonotic disease (animal-to-human transmission) caused by the intracellular bacteria of the species *Coxiella burnetii* [1,2,3,4,5]. This microorganism was initially classified within the genus *Rickettsia* (*R. burnetii*, *R. diaporica*). However, the detection of differential characteristics led to its classification as the only species of the genus (*Coxiella*) within the family *Rickettsiaceae* and order *Rickettsiales*. As a result of genetic analysis (mRNA and complete genome sequencing of *C. burnetii*), the microorganism has now been reclassified within the phylum Proteobacteria, gamma subdivision, the family Coxiellaceae, and the order *Legionellales* [6], which explains the serologic cross-reactivity between *C. burnetii* and other species (e.g., of the genus *Legionella*).

Until relatively recently, the genus *Coxiella* included only *C. burnetii*. About a decade ago, another species (*Candidatus* Coxiella massiliensis) was identified, although there are few references. This new species has been associated with a different clinical picture known as SENLAT (scalp eschar and neck lymphadenopathy after tick bite) [7].

**Figure 1:**
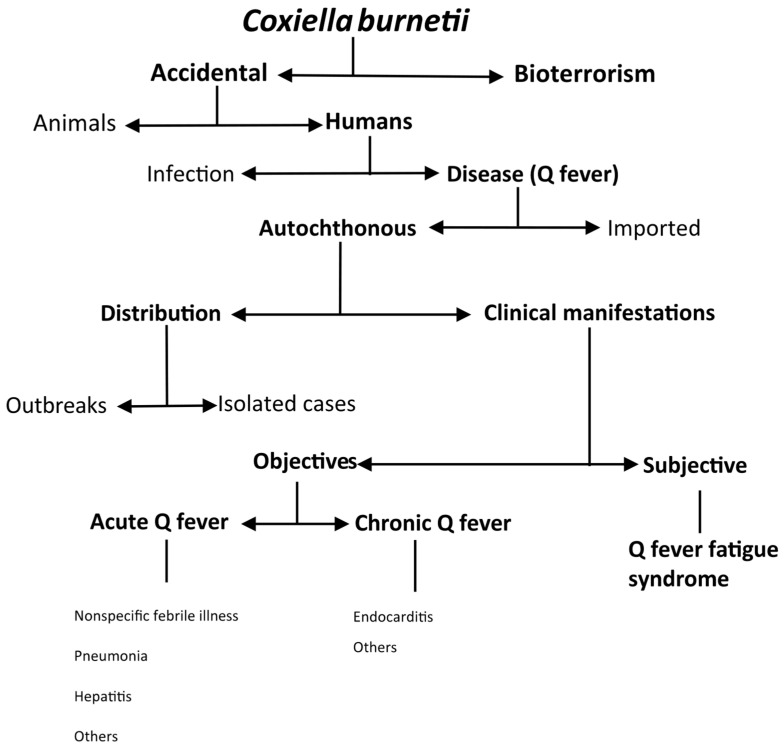
Epidemiologic patterns of *Coxiella burnetii* infection.

*C. burnetii* infection is a zoonosis with worldwide distribution that affects both humans and animals [2]. It is of significant public health concern because of its very diverse clinical and epidemiological patterns (Figure 1).

The most common form of infection is accidental. Nevertheless, due to the characteristics of *C. burnetii* (see below), such as its high infectiousness and resistance to environmental agents, the CDC has classified it as a category B potential bioterrorism agent [1,2]. In domestic ruminants, *Coxiella burnetii* infection can cause reproductive disorders such as infertility, stillbirth, abortion, endometritis, and mastitis in pregnant animals, but it tends to be asymptomatic in non-pregnant animals [2]. In humans, infection may be asymptomatic or cause disease (Q fever). In an outbreak reported in Switzerland, only 50% of people who seroconverted had clinical manifestations [8]. However, in the Canary Islands, up to 21.5% of the population had serological evidence of *C. burnetii* infection, in contrast to a much lower number of diagnosed cases of Q fever [9,10]. Most infections are acquired autochthonously, although due to its worldwide distribution, cases of imported disease have also been documented in travelers [11,12,13]. Autochthonous Q fever can occur in outbreaks [14] or sporadically [3,15]. The two main clinical presentations of Q fever are acute (i.e., nonspecific febrile illness, pneumonia, or hepatitis) and chronic (i.e., endocarditis). A complication of Q fever is post-Q fever fatigue syndrome, whose manifestations are subjective [1,2,3].

Although Q fever has a worldwide distribution [2,3], the published data are variable. The reasons for this include the following: (*i*) the different epidemiological forms of infection; (*ii*) the variable clinical manifestations of the disease; (*iii*) the lack of mandatory reporting of Q fever in many countries; and (*iv*) interest in studying this entity since, as stated by D. Raoult, “*differences in prevalence often reflect differences in interest in studying the entity*”.

As with many infections, the outcome depends on the microorganism, the form of infection, and the host characteristics. This review briefly discusses the various aspects of the pathogenesis (causative agent and mechanisms of injury) and pathophysiology (consequences and manifestations) of Q fever in humans.

## 2. Microbiology

**Figure 2:**
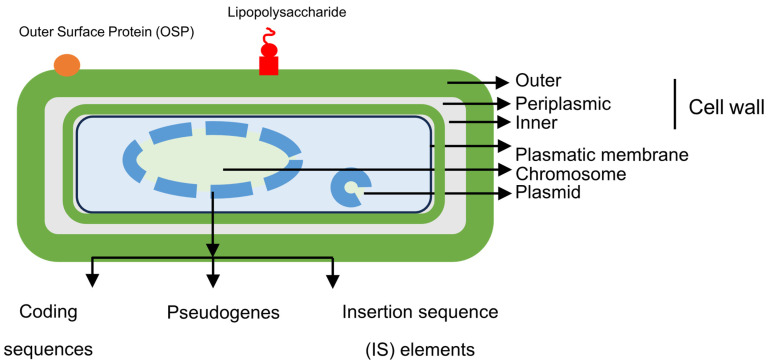
Structural features of *Coxiella burnetii*.

*Coxiella burnetii* is a small, immotile, obligate intracellular coccobacillus, although it has a membrane similar to that of Gram-negative bacilli (Figure 2).

Nevertheless, *C. burnetii* does not stain well with the Gram stain. The main techniques used for microscopic visualization in tissues are the Giemsa, Giménez, or Machiavello stains [2]. This bacterium produces two morphologically and functionally distinct strains, SCV (*small cell variants*) and LCV (*long cell variants*) [16], which can be separated for analysis by cesium chloride gradients. While both forms are capable of infecting eukaryotic cells, they have important structural and functional differences [16,17,18,19] (Table 1).

These structural characteristics give *Coxiella burnetii* an extraordinary capacity for extracellular survival and resistance to physical or chemical agents. It can survive for 7–10 months on wool at 15–20 °C, for over one month in fresh meat stored in cold storage, and for more than 40 months in skimmed milk at 4–6 °C [2]. At the same time, viable microorganisms have been recovered after heating at 63 °C for 30 min, after exposure to a 10% saline solution for 180 days, exposure to 0.5% formalin, or sonication in distilled water for more than 30 min [16].

### 2.1. Essential Structures

Essential structures of *C. burnetii* include some components of the outer membrane (OMPs, outer membrane proteins, and LPS, lypopolysaccharide), peptidoglycan, and genetic material (bacterial chromosome and plasmids).

*OMPs* are a group of molecules found only in the outer membranes of Gram-negative bacteria, as well as in mitochondria and chloroplasts. More specifically, OMPa plays an important role in the adhesion-mediated internalization of *Coxiella burnetii* by non-phagocytic cells [5,17,20] (see below).

*Lipopolysaccharides* are molecules found in the outer membrane of Gram-negative bacteria and consist of three main components, lipid A (inserted into the membrane), a core oligosaccharide, and a distal polysaccharide (antigen O). *C. burnetii* expresses LPS, although its endotoxic capacity is low [21], approximately 1000 times lower than other bacteria such as *Escherichia coli* [17]. In the biological cycle of *Coxiella burnetii*, a critical aspect of virulence is due to structural modifications in the LPS known as phase variation. In nature, *Coxiella burnetti* expresses phase I antigens that correspond to LPS with complete O side chains (or smooth LPS), which can mask the presence of surface antigens. After subculture in cell media or during the course of disease in the host, LPS is transformed into phase II antigens (with a truncated O side chain), which do not restrict antibody access to the bacterial surface. Phase II microorganisms are therefore less virulent. Studying the antibodies against both types of antigen is important for diagnosis (see below). It should be noted that these two concepts of bacterial size (SCV or LCV) and LPS structure (phase I or phase II), while related, are not equivalent, and they should not be confused [22].

*Peptidoglycan*, especially in SCVs, has a high number of cross-links [3] and, together with its associated proteins, is an important factor in resistance to both environmental conditions and the intracellular digestion of *C. burnetii* by eukaryotic cells (lysozyme) [23].

The *genome* of *Coxiella burnetii* consists of a bacterial chromosome and facultative plasmids.

The *bacterial chromosome*, which has been characterized in various studies, is circular; although, previous work has suggested the possibility of a linear structure [6,24,25]. The size ranges from 1.5 to 2.4 M base pairs (2.1 in the Nine Mile reference strain), with a G+C content of 42.6%. It has a smaller genome than extracellular bacteria, but it is considerably larger than that of other intracellular bacteria. It contains three types of structures, coding genes, pseudogenes, and insertion sequences (Figure 2). The number of coding genes exceeds 2000 and includes the following: (*i*) Na^+^/H^+^ exchangers involved in pH homeostasis and survival in acidic media; (*ii*) osmoprotective transporters; (*iii*) drug efflux systems that facilitate resistance within the phagolysosome; (*iv*) carbohydrate transporters (xylose and glucose); (*v*) transporters of amino acids and peptides; (*vi*) enzymes involved in the Embden–Meyerhof pathway (glycolysis) and the Entner–Doudoroff pathway (an alternative pathway that catabolizes glucose to pyruvate) in gluconeogenesis, the electron transport chain, pentose pathway, and tricarboxylic acid pathway; (*vii*) enzymes involved in the synthesis of purines, pyrimidines, fatty acids, or phospholipids; and (*viii*) ankyrin domain proteins related to bacterial adhesion to host cells. Unlike other obligate intracellular bacteria, in which there is usually little opportunity for genetic exchange, the different variants of *Coxiella burnetii* have between 30 and 59 insertion sequences (IS), which represents high genomic plasticity. On the other hand, genomic analysis has revealed a very high number of pseudogenes, which suggests that the microorganism is in an early stage of adaptation to the host.

The following four types of *plasmids* have been described in different isolates of *Coxiella burnetii*: QpH1, QpRS, QpDG, and QpDV [26,27,28,29,30]. A sequence homologous to these plasmids integrated into the bacterial chromosome of some genetic variants (denoted as plasmidless or “S”) has also been described. In cross-hybridization studies, a conserved part (the core), representing approximately 50% of the genetic information of all the plasmids or pseudoplasmids indicated, has been observed. Samuel et al. first described the 37 kb QpH1 plasmid in 1983 in the Nine Mile strain, which was obtained from ticks [26]. The same group found the QpRS plasmid, larger in size (39 kb), two years later in a *Coxiella burnetii* (Priscilla) strain from goat placenta [27]. The QpDG plasmid, identified in 1989 by Mallavia et al. in wild rodents from Utah (Dugway), is the largest plasmid (42 kb) [28]. Finally, in 1995, QpDV, the smallest plasmid (33.5 kb), was identified in *Coxiella burnetii* strains; two were Russian strains (pneumonia in one patient, cow’s milk in another), and three were French (acute Q fever, aortic aneurysm, and chronic endocarditis) [29]. From the point of view of bacterial pathogenesis, plasmids play an important role by carrying genes encoding, among others, substrates for the type IV secretion system (T4SS), an important virulence factor (see below) [31]. Although the four plasmids mentioned here belong to different genetic groups (see below) and have been associated with different clinical forms of Q fever, the results are inconclusive [27].

### 2.2. Coxiella Burnetii Genotypes

Using various molecular biology techniques, *Coxiella burnetii* has been found to exhibit significant genetic variability [32]. The techniques used for genotyping include restriction fragment length polymorphism (RFLP) [33], pulsed field gel electrophoresis (PFGE) [34], multispacer sequence typing (MST) [35,36], variable number tandem repeat (VNTR) analysis [37], multiple-locus variable-number of tandem repeat analysis (MLVA) [38,39], and single nucleotide polymorphism (SNP)-based typing [40,41], as well as different restriction enzymes and genetic material from humans, other mammals, and ticks.

There is a high number of specific genotypes, which varies according to the technique used. The number of genomic groups, also variable, is lower. These genotypes have been associated with more or less virulent forms, different geographical distributions, different hosts, and different clinical forms (acute or chronic), although the results have not always been clear [2,17,35,42,43]. In the search for genetic markers associated with specific clinical manifestations of Q fever, the expression of molecules such as adaA (acute disease antigen) has been studied. adaA expression has been associated with acute Q fever, and its absence has been associated with chronic Q fever [44], although later studies have not confirmed this association [45].

In Spain, Jado et al. [46] studied *Coxiella burnetii* genotypes using PCR, with primers described by Beare [47], and adaA expression [44]. The results indicated that the most common genomic groups were GG-IV in humans, GG-III in livestock, and GG-VII and GG-IV in ticks [46].

## 3. Pathogenesis

**Figure 3:**
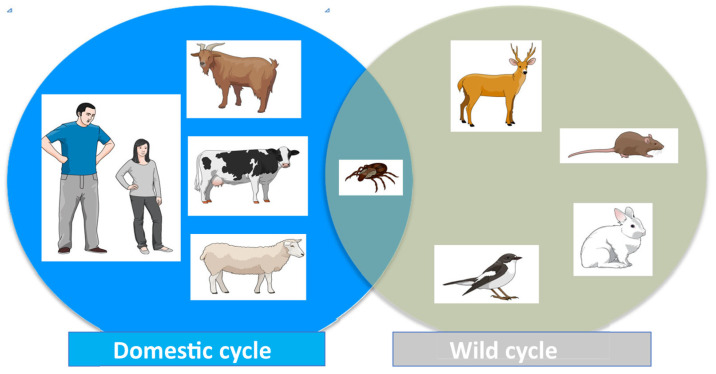
Cycle of *Coxiella burnetii* infection.

*C. burnetii* infection is an anthropozoonosis primarily transmitted from an animal reservoir to humans. In the epidemiology of Q fever, it is important to distinguish between two main interconnected types of reservoir and/or sources of infection (Figure 3) [1,2,3,48,49]. The first includes domestic or peridomestic animals and consists mainly of goats, sheep or cattle, and, to a lesser extent, cats and dogs (domestic cycle). The second reservoir would include rodents and small mammals but also occasionally birds, reptiles, amphibians, and fish (wild cycle). Ticks play a significant role as vectors for the transmission of *C. burnetii* during the wild cycle and for its entry to the domestic cycle (see below) (Figure 3).

### 3.1. Routes of Infection

The infection of humans usually occurs through contact with biological products of domestic animals (especially birth byproducts but also milk, feces, and urine) [1]. The animals involved in this form of transmission vary considerably according to geographical area (i.e., sheep in Canada, cattle in Japan, goats in the USA) [1]. In our region (the Canary Islands, Europe), the overall seroprevalence in 2010 was 34.7% (60.4%, 31.7%, and 12.2% in goats, sheep, and cattle, respectively). The most affected regions in the island of Gran Canaria were those where small ruminants, particularly goats, were reared (south and east of the island), while the northern area, where cattle are concentrated, showed the lowest prevalence [50].

**Figure 4:**
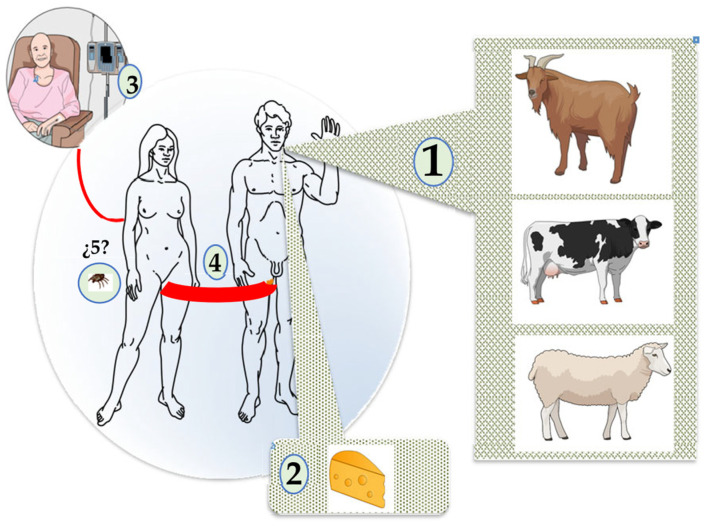
Human infection by *Coxiella burnetii.* 1. Airborne. 2. Consumption of milk and its derivatives 3. Transfusion. 4. Sexual. 5. Ticks.

In humans, airborne transmission is the primary mode of *Coxiella burnetii* infection, although other less important routes have been described, such as the consumption of dairy products, with human-to-human transmission and tick bites being exceptional [1,2,3,48,49] (Figure 4).

Airborne transmission is significant in outbreaks [14] and for professionals in contact with animals (farmers, slaughterhouse workers, veterinarians) or human biological samples (microbiologists, pathologists) [2]. It should be noted that the lower respiratory tract is the primary site of infection; in fact, in experimental studies, the introduction of *Coxiella burnetii* into the upper airways was shown to be a rare form of infection [2]. Despite this, *Coxiella burnetii* infection (and/or disease) is frequently diagnosed in people with no epidemiological evidence of contact with domestic animals. In this sense, the structural characteristics of *C. burnetii* (its small size, ability to be carried by the wind in dust) allow it to spread up to 30 km from the source of the infection, depending on the direction and intensity of the wind [51,52].

The consumption of dairy foods has traditionally been considered a route of *C. burnetii* infection [2]. However, the following two points should be taken into account: (*i*) the consumption of contaminated food is not synonymous with transmission through the digestive tract, and (*ii*) the epidemiological data are inconclusive; in some series of patients, the consumption of raw milk and its derivatives is more frequently associated with *C. burnetii* infection than pasteurized milk, while in other series, the data are not confirmed [53,54].

Human-to-human transmission is very rare, although there have been reports of both transfusion-related and sexual transmission [55,56].

The role of ticks in *C. burnetii* infection is complex. This pathogen was first detected in 1935 (then called *Rickettsia diasporica*) [57,58]. The classic studies (included in ref. [2]) have described many interesting aspects, including the following: (*i*) infection has been reported in both hard ticks (i.e., *Amblyomma* spp., *Dermacentor* spp., *Haemaphysalis* spp., *Hyalomma* spp., *Ixodes* spp., and *Rhipicephalus* spp.) and soft ticks (i.e., *Argas* spp. or *Ornithodoros* spp.); (*ii*) ticks can acquire *C. burnetii* by hematophagy after ingesting blood from infected mammals, although the efficiency of transmission is highly variable, depending on the species; (*iii*) while *C. burnetii* can infect ticks, it does not necessarily change their vital activity or survival; (*iv*) *C. burnetii* reproduces in the digestive tract, can persist for a long time (from months to years), and is excreted in feces and/or coxal fluid; and (*v*) transstadial and transovarial transmission of *C. burnetii* has been demonstrated in several tick species. Our group has studied the presence of *C. burnetii* infection in many ticks (1738) in the Canary Islands [59]. Samples were collected from vegetation (67%, mostly *Hyalomma lusitanicum*), livestock (19%, mainly *Riphicephalus turanicus*), domestic dogs (10%, *Riphicephalus sanguineus* and *Riphicephalus turanicus*), and wild animals (4%, mainly *Hyalomma lusitanicum*). *C. burnetii* DNA was detected in 11.3% of livestock ticks, 6.9% of dog ticks, 6% of wild animal ticks, but not in ticks obtained from vegetation. However, human infection directly associated with tick bites is exceptional [60].

Anecdotally, *C. burnetii* infection in free-living amoebae and its possible role in an air-conditioning-related outbreak has also been documented [61,62].

### 3.2. General Aspects of Pathogenesis

There is an extensive literature on pathogenic aspects of *Coxiella burnetii* infection, although the data are sometimes confusing. The data are listed as follows.

(*i*) Several experimental models of *C. burnetii* infection have been described, mainly in mice, guinea pigs, and non-human primates [63,64,65,66,67,68]. The results varied according to the type of animal studied, the route of administration of *Coxiella burnetii* (intraperitoneal, intratracheal, inhalation), and other factors (e.g., immunosuppression, genetic modification). Several syngeneic *mouse* species (particularly As/J and BALBc) were susceptible to *Coxiella burnetii* by both inhalation and intraperitoneal routes. These species have also been used in other Q fever models in pregnant BALB/c mice or BALB/c mice treated with cyclophosphamide that developed endocarditis. On the other hand, several mouse models have elucidated important aspects of the pathogenesis of Q fever, such as mice with SCID (severe combined immunodeficiency) strains that developed endocarditis with focal calcifications very similar to those seen in humans, mice overexpressing IL-10 with reduced granuloma formation and accumulation of *Coxiella burnetii* in tissues, and knockout mice lacking TLR-2 or IFN-γ that exhibited a febrile response to *Coxiella burnetii* phase II. Finally, manipulating different subtypes of B lymphocytes, CD4 T cells, and CD8 T cells in SCID or wild-type mice has established the role of these cell lines in the pathogenesis of Q fever. The *guinea pig* is another micromammal that has been used in experimental studies. Infection with *Coxiella burnetii* by the intraperitoneal or inhalation route produced a picture of infection similar to that seen in humans. In addition, an aortic valve injury prior to *Coxiella burnetii* infection triggered endocarditis, albeit with more acute features. Although few studies have been performed, the primate model of Q fever, particularly in *macaques* (*M. fascicularis* and *M. mulatta*) is the one that most closely resembles human infection. However, experimental data cannot always be extrapolated to those obtained in humans.

(*ii*) Coxiella burnetii can penetrate multiple cell lines, including cells of the phagocytic mononuclear system (macrophages and monocytes), as well as epithelial and endothelial cells [17], although the specific mechanisms of entry differ depending on the cell lineage (macrophages or monocytes) [17]. Traditionally, *Coxiella burnetii* culture required cell media with strict isolation conditions (P3). Recently, axenic media have been developed that enable *Coxiella burnetii* to grow outside host cells. The media are designed to mimic the characteristics of the *Coxiella*-containing vacuole (CCV), within which the bacterium replicates, as do the metabolic requirements that enable it to survive [69]. For culturing *Coxiella burnetii* in axenic media, the following are required: a moderately acidic pH (pH ≈ 5) maintained by a citrate buffer, concentrations of Na^+^, K^+^, and Cl^−^ ions similar to those of the extracellular medium, peptides as the primary source (preferably carbon over glucides) in the form of neopeptone, casamino acids, and L-cysteine, and a low oxygen concentration (1–5%).

(*iii*) The nomenclature used in the literature can also be confusing. It is important to note that the main form of cellular entry of *Coxiella burnetii* is endocytosis. This process involves the internalization of extracellular material surrounded by the plasma membrane, from which it is subsequently shed to form endosomes. Depending on the size of the exogenous material, the following two forms can be distinguished: phagocytosis (large size), which triggers the formation of pseudopods, and pinocytosis (small size). In this context, endosomes and phagosomes can be used as synonyms. In some publications, *Coxiella*-containing vacuole (CCV) is used as a synonym of endosome [70]. Endosomes are classified according to their location, the presence of membrane-bound GTPases (such as Rab), other proteins (e.g., *Coxiella* vacuolar proteins or Cvp), as well as the lipid composition of their membranes [70]. For example, early endosomes are formed by the convergence and fusion of endocytosis vesicles, are located at the cell periphery close to the plasma membrane, are characterized by the presence of Rab5 and CvpB proteins in their membranes, and are rich in phosphatidyl inositol triphosphate [PI(3)P]. In contrast, late endosomes, also known as multivesicular bodies (MVBs), are located in deep intracellular zones, express Rab7 and CvpA, and are rich in phosphatidyl inositol diphosphate [PI(3,5)P2].

### 3.3. Stages of Infection

#### 3.3.1. Entry of *Coxiella burnetii* by the Respiratory Route

**Figure 5:**
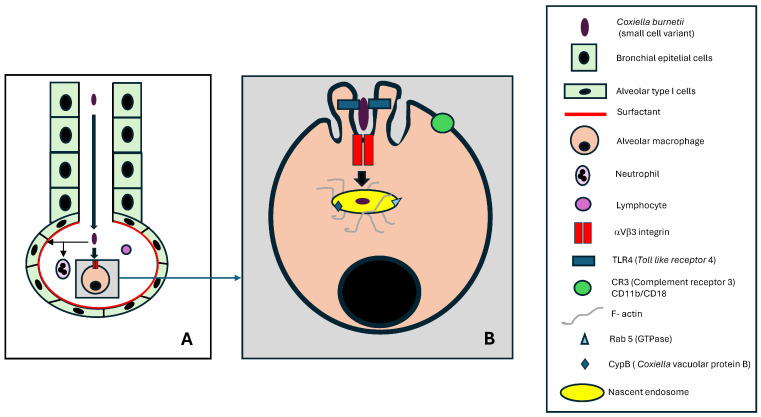
Entry of *Coxiella burnetii* by the respiratory route: (**A**) lower respiratory tract, (**B**) alveolar macrophage.

In humans, the respiratory tract is the primary route of penetration for *Coxiella burnetii* (Figure 5A). The small size and structural characteristics of the microorganism enable direct access to the alveolar region (detection studies in the upper respiratory tract are often unsuccessful in patients with Q fever). On the other hand, lower expression of aVb3 integrins in the bronchial (and alveolar) epithelium may limit the adhesion of the SCV of *Coxiella burnetii* to host cells [17].

The alveolar region is lined by type I pneumocytes (and to a lesser extent, type II pneumocytes) coated with surfactant. An interesting feature is that *C. burnetii* can bind to surfactant protein D, potentially restricting its contact with alveolar macrophages (AM) [21,71].

The cell population of the alveolar lumen consists mainly of alveolar macrophages (about 90%), followed by lymphocytes (about 9%) and neutrophilic polymorphonuclear leukocytes, or neutrophils (around 1%) [72]. Although it has been observed that neutrophils can be infected by *C. burnetii*, they cannot destroy this bacterium [21]. Alveolar macrophages are the primary cells involved in the initial entry and infection of *C. burnetii* in humans.

Alveolar macrophages (AM) are mainly derived from monocytes that enter the alveolar region through the bloodstream, although local proliferation has also been shown to play a role [72,73]. These macrophages show important differences from interstitial macrophages, another local population of cells of the mononuclear phagocyte system (MPS) [73]. AMs exhibit low CD11b and high CD11c expression, the opposite of the interstitial macrophages [73], which is important in the pathogenesis of Q fever.

#### 3.3.2. Penetration of Alveolar Macrophages by *C. burnetii*

The infection of alveolar macrophages by *C. burnetii* involves first the adhesion of the bacterium, followed by its internalization [5,17,42,74,75,76] (Figure 5B). The following two key alveolar macrophage molecules are involved in bacterial adhesion: TLR4, essential in lipopolysaccharide recognition, and αVβ3, which acts as a receptor. The adhesion process is associated with morphological changes (visible by electron microscopy) of the AM membrane in the form of protrusions (membrane ruffling) that exclude from the contact zone other molecules such as CR3 (CD11b/CD18) or TLR2 that would be deleterious to the survival of *C. burnetii*.

Essential to this process is the reorganization of the F-actin cytoskeleton, which contributes to the stability of the nascent endosome and provides a track for vesicular fusion activities. The main molecules involved in signaling are RhoA and ROCK.

The nascent endosome formed as a result of these processes has a membrane rich in lipids, such as phosphatidylinositol-3-phosphate (PI[3]P) and phosphatidylserine, and proteins such as CvpB (involved in binding to the phospholipids crucial for microtubule-associated protein 1A/1B-light chain 3 (LC3) in autophagy) and rab5 (which stimulates early endosome fusion). The interior of the nascent endosome maintains a pH of approximately 6.0 and contains *Coxiella burnetii* in its SCV form.

#### 3.3.3. Life Cycle of *Coxiella burnetii* Within the Alveolar Macrophage 

**Figure 6:**
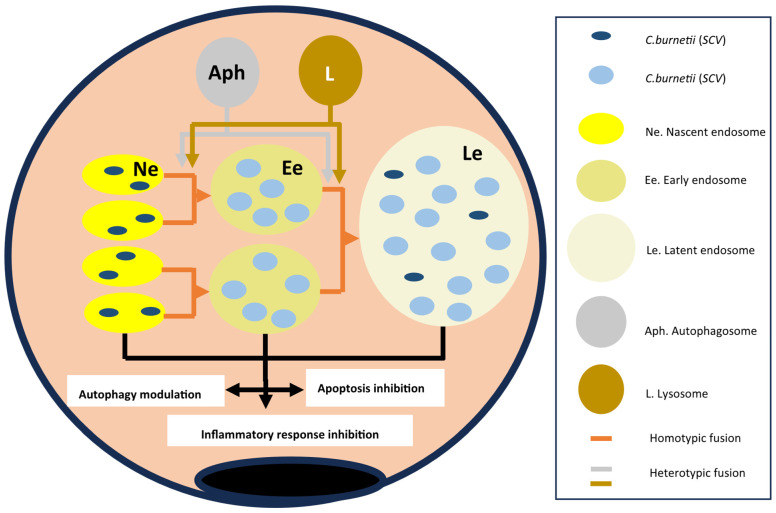
Life cycle of *Coxiella burnetii* within the alveolar macrophage. Flesh color lines indicate the origin of the molecules involved (blue and brown) and the evolution of the endosomes (orange). The black lines indicate the consequences on the alveolar macrophage.

*C burnetii* develops inside an alveolar macrophage within vacuoles formed by individual endosomes merging (homotypic fusion) or attaching to other subcellular structures (autophagosomes or lysosomes) [5,42,77] (Figure 6).

**Figure 7:**
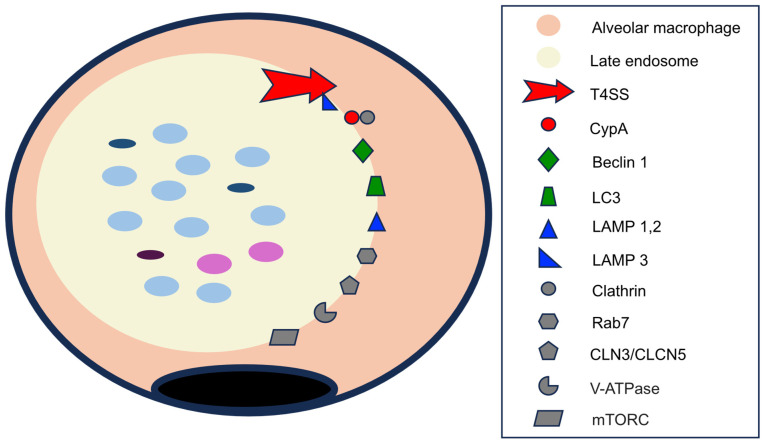
Membrane proteins in the late endosome. Proteins of *C. burnetii* origin are indicated in **red**; autophagosome membrane proteins are in **green**; lysosomal membrane proteins are in **blue**; and cytoplasmic or those shared by several organelles are in **gray**. See text for abbreviations.

The evolution of endosomes from nascent to late/multivesicular forms depends on molecules derived from *Coxiella burnetii* and those derived from the cytoplasm and organelles of the alveolar macrophage. The different types of endosomes differ in structure and functions in the following ways:

- *Membrane lipids*. Like most biological membranes, the membrane of endosomes containing *C. burnetii* consists of cholesterol and phospholipids. The cholesterol content is exceptionally high (comparable to that of the plasma membrane) due to the microorganism’s induction of cholesterol synthesis [17,78]. However, the major type of phospholipid varies between the early [PI(3)P] and late [PI (3,5) P2] endosomes.
- *Membrane proteins*. A very high number of proteins have been described in the CCV membrane. The key proteins are shown in Figure 7.

Among the essential proteins derived from *C. burnetii* are those of the multi-protein complex Dot/Icm (defective in organelle trafficking/intracellular multiplication) system, a type IVB (TSS4) secretion system [21,39,42,78], responsible for the translocation of many proteins from the endosomes to the macrophage cytoplasm [39,42,78]. The different molecules delivered to the cytoplasm are involved in endosome formation and development, autophagocytosis, the inhibition of apoptosis, and the modification of the macrophage inflammatory response. An important group of proteins transferred by the TSS4 are those included in the Cvp (*Coxiella* vacuolar protein) family of effector proteins, of which six types (A–F) have been described [42]. The function of CvpB in the nascent endosome has been described previously [5,21,70,74]. CvpA is expressed at later stages of endosome development, and it exerts its effects by interacting with the adaptor-related protein complex 2 (AP2) and binding to the cytoplasmic protein clathrin [70]. The main effects of CvpA are vacuole expansion, vesicular trafficking, and fusion of autophagosomes [42,74]. Although less well known, CvpC is also involved in autophagy [5].

A second group of endosomal membrane proteins are derived from fusion with autophagosomes, the best characterized being beclin 1 and LC3 [42]. Beclin 1 is a key molecule in vacuolar development, the regulation of autophagy, and the inhibition of apoptosis (see below) [79].

Lysosomes contribute several types of membrane proteins to CCVs, of which the best characterized is the LAMP (lysosome-associated membrane glycoproteins) family. LAMP 1–3 molecules are the best characterized [5,17], with LAMP1 and LAMP2 being important in CCV acidification and autophagy stimulation [80] and LAMP3 showing importance in TTS4 stabilization [81].

Finally, other cytoplasmic proteins, including those shared with other organelles, are localized on the membrane of the CCV itself. These include the following: (*i*) pH regulatory proteins encoded by CLN3 and CLCN5 genes (neuronal ceroid lipofuscinosis type 3 and type 5), which are involved in CCV acidification [74]; (*ii*) vacuolar ATPases, which contribute to lowering the pH of endosomes [17,82]; (*iii*) rab7, a member of the GTPase family, which regulates autophagy by participating in CCV formation and maturation [70,75]; (*iv*) clathrin, a protein involved in the formation of clathrin-coated vesicles, which are related to autophagocytosis [74]; and (*v*) mTORC (mammalian target of rapamycin complex 1, or mTORC1), which inhibits autophagy [5].

*Content*. For *Coxiella burnetii* proliferation and activation within the late endosomes of host cells, maintaining a microaerophilic environment and a low pH (around 4.0–4.5) is essential [17,74,75]. Several of the molecules mentioned above are involved in maintaining the low pH, including vacuolar ATPase, CLN3, CLCN5, LAMP1, and LAMP2. One major consequence of the acidic pH is that it reduces the effectiveness of many antimicrobials in killing *C. burnetii* residing within this cellular compartment [75,76].

Several enzymes such as cathepsin B, cathepsin D, and lysosomal acid phosphatase (ACP2) can be detected within late endosomes because they bind to and are transported within lysosomes [17,42].

With respect to *Coxiella burnetii*, a process of bacterial proliferation and transition between nascent and late endosomes takes place [5,17,21] (Figure 7). The bacteria replicate primarily within the nascent endosomes, which are the main site of the SCV form. Within hours, the SCVs transform into LCV forms, which constitute practically 100% of the bacterial population. In later stages, a portion of the bacteria revert to their SCV forms.

#### 3.3.4. Effects of *Coxiella burnetii* Infection of Alveolar Macrophages

The functional changes that take place within the alveolar macrophage facilitate *Coxiella burnetii* proliferation and survival. Several complementary mechanisms are involved, including the modification of autophagy, the inhibition of apoptosis combined with the promotion of survival signals, and the inhibition of the inflammatory response.

*Modification of autophagy* [83,84]. Autophagy is an important, highly regulated biological process in which double-membrane organelles called autophagosomes are formed that sequester damaged subcellular structures and intracellular bacteria for degradation. In certain infections (such as *Salmonella* spp., *Legionella* spp., and *Mycobactrieum tuberculosis*), autophagy restricts bacterial proliferation and limits the severity of the infection. However, there are both direct and indirect data that *Coxiella burnetii* stimulates autophagy by increasing bacterial proliferation and CCV size. Some molecules involved in this phenomenon are beclin, LC3, LAMP1 and 2, several CYP proteins, and Rab7. Proteins derived from *C burnetii* that inhibit mTOR have also been described, although they have not been characterized.

*Inhibition of apoptosis.* Apoptosis is a form of programmed cell death characterized by specific morphological and functional changes [85]. The final common pathway of apoptosis is mediated by enzymes called caspases (cytosolic aspartate-specific proteases). The common final pathway (or execution pathway) depends on caspase 3 and, to a lesser extent, caspase 6 and 7, which are activated through the following three initiating pathways: the mitochondrial (or intrinsic) pathway, the lethal receptor (extrinsic) pathway, and the direct pathway, respectively. The key event of the mitochondrial pathway is the formation of the apoptosome complex, initiated when cytochrome c released from the mitochondria binds to the adaptor protein (APAF-1: apoptotic protease-activating factor 1), along with dATP, and activates procaspase 9, followed by caspase 9, which ultimately triggers the final common pathway. This pathway is regulated by many anti-apoptotic molecules (such as Bcl-2 and Bcl-xl) and pro-apoptotic molecules (such as Bad, Bax, or p32). The lethal receptor pathway is initiated by the binding of extracellular ligands (such as tumor necrosis factor α [TNF-α] in soluble or membrane-bound form or FasL) to cellular death receptors. Various types of adaptor proteins activate caspase-8, which also activates the common pathway. The direct apoptosis pathway derives from the activation of caspase 3, which is triggered by granzymes entering target cells through pores induced by cytoperforins and specific receptors.

**Figure 8:**
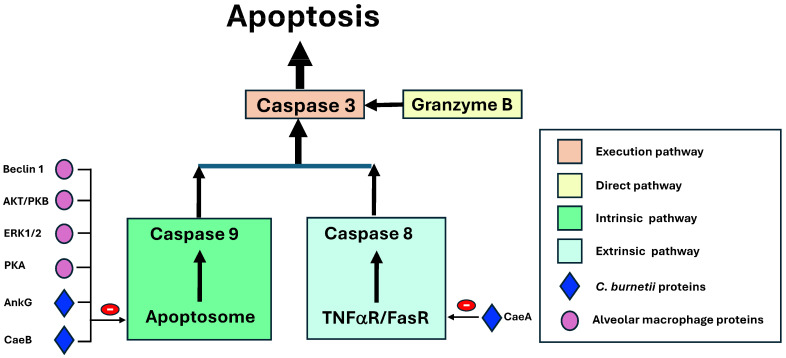
The inhibition of alveolar macrophage apoptosis by *Coxiella burnetii.* AKT/PKB: Protein kinase B; AnkG: Ankyrin G; Cae A /CaeB: *C. burnetii* anti-apoptotic effectors; ERK 1/2: extracellular signal-regulated kinases; PKA: Protein kinase A. Color indicates the pathways and arrows stimulant or inhibitory factors.

*Coxiella burnetii* infection inhibits cell apoptosis through multiple mechanisms and is mediated directly by effector proteins secreted (via the TSS4) by the bacterium and by modulating macrophage responses to bacterial products. The effector proteins inhibit the major caspases (i.e., 3 and 9) and upregulate the inhibitors of these enzymes [5,86]. Most apoptosis inhibitors target the intrinsic pathway (Figure 8) [5,17,21,39,42,78,86,87,88]. Of the macrophage proteins, beclin 1 activates bcl2 [17,42]; AKT/PKB (protein kinase B) inhibits both Bad and Bax, while also activating pro-apoptotic factors such as FOXO (forkhead box O) [42,78,86,87]; ERK1/2 (extracellular signal-regulated kinases) degrade Bad [42,78,86]; and PKA (protein kinase A) inhibits Bad [42]. On the other hand, several effector proteins secreted by *C. burnetii* have an anti-apoptotic action on this pathway. These include AnkG (ankyrin G), which inhibits p32 [39,88] and CaeB (*C. burnetii* anti-apoptotic effector B) [21,39,88]. Less common mechanisms include the inhibition of the extrinsic pathway by CaeA (*C. burnetii* anti-apoptotic effector A) [21] and the direct pathway [89].

#### 3.3.5. Inhibition of the Inflammatory Response

Cells of the phagocytic mononuclear system (macrophages and monocytes) can be polarized into two major polarization phenotypes, M1 and M2 [5,73]. The M1 phenotypic profile is proinflammatory, with the activation of bacterial killing mechanisms (reactive oxygen species (ROS) and nitric oxide), the formation of inflammasomes, the production of several cytokines such as IL-1β or TNFa, and the expression of certain membrane molecules (such as TLR-2, CD80, CCR7) [5,90]. In contrast, the M2 phenotype exhibits an anti-inflammatory profile with the inhibition of bacterial killing mechanisms, the inflammasome formation, the synthesis of a different group of cytokines (IL-10 or TGFß), and other membrane molecules (i.e., CCL18, mannose receptor) [5,90].

The infection of alveolar macrophages by *C. burnetii* has multiple effects on their inflammatory response. Bacterial killing is limited since *C. burnetii* is inherently resistant to digestion by lysosomal hydrolases [42], ROS generation is reduced [4], and arginase 1 limits nitric oxide production [90]. On the other hand, macrophage polarization towards the M2 phenotype occurs, inhibiting the expression of genes associated with M1, except for IL-6 [5,75,90,91]. Finally, there is non-canonical inhibition of inflammasome formation [74], partly due to the inhibition of caspase 11 activation by the bacterial molecule IcaA (inhibition of caspase activation) [21].

The genesis of these processes involves, among other things, excluding TLR2 from bacterial adhesion [92] and inhibiting the NF-κB pathway [74].

#### 3.3.6. Systemic Dissemination of *Coxiella burnetii*

Alveolar macrophages infected with *C. burnetii* exhibit a phenotype characterized by the stimulation of autophagy, the inhibition of apoptosis, and the inhibition of the inflammatory response. *C. burnetii* can exit the AM by the following three different mechanisms: the endosomal and plasma membranes rupture and release the bacteria; exocytosis, by cell-to-cell contact; and extrusion in vesicles containing the bacteria [5].

The most common fate of alveolar macrophages and their derived products is clearance via respiratory secretions that ascend to the bronchial region [72]. However, they can also reach other parts of the body via the lymphatic system and the bloodstream [5,72]. In *C. burnetii* infection, evidence of blood dissemination is found by detecting its genetic material, although this is limited to the acute phase and usually decreases after antibacterial treatment [21,93].

Dissemination allows *C. burnetii* to infect many cell lineages (such as those within the phagocytic mononuclear system (monocytes and macrophages), dendritic cells, epithelia, parenchyma, and fibroblasts) [42,94]. One aspect that differentiates the interaction between *C. burnetii* and other phagocytes is that adhesion and internalization of the bacterium process by other phagocytes is primarily mediated by CR3 and TLR2 (αVβ3 integrin is expressed at low levels on resting monocytes) [17], while, in non-phagocytic cells, OMPa plays a crucial role [5,17,20].

### 3.4. Systemic Immune Response

The outcome of systemic *C. burnetii* infection is influenced by bacterial factors (genotypes) [78,95] and the host immune response. There is some evidence for the involvement of innate immunity, the acquired humoral response, and cell-mediated immunity in the pathogenesis of *C. burnetii* infection [17], although there are significant differences depending on the clinical manifestations (see below) and sometimes conflicting data [21,90,94,96].

#### 3.4.1. Asymptomatic *C. burnetii* Infection

For obvious reasons, there is little information on the immune response in asymptomatic individuals, although the presence of antibodies against phase II antigens suggests, at least in part, the involvement of humoral immunity.

#### 3.4.2. Acute Q Fever

Several components of innate immunity play an important role in the pathogenesis of acute Q fever. The most important membrane molecules are TLR2 and TLR1, which, when inhibited, reduce cytokine production by phagocytes [21,95,97,98] and TLR10, which has the opposite effect. Intracellular NOD (nucleotide-binding oligomerization domain) also plays an important role in cytokine synthesis in acute Q fever [21,95]. Finally, *C. burnetii* can activate plasmacytoid dendritic cells to stimulate the production of type I interferons, which then upregulate genes that encode pro-inflammatory cytokines [21,99].

During acute Q fever, the humoral immune response is characterized by the synthesis of antibodies that primarily target phase II antigens; later, and to a lesser extent, antibodies against phase I antigens may also develop. This explains the predominant release of LCVs from the alveolar region (see above) and the slower processing of LCVs by phagocytic cells [76]. In some cases, IL-6 production by T lymphocytes [90] can also stimulate the synthesis of autoantibodies [100] (see below).

The cellular immune response is very important in the pathogenesis of acute Q fever and involves both phagocytic cells and lymphocytes. Phagocytic cells stimulated by *C. burnetii* exhibit an M1 phenotype, with the production of **TNFα** and IL-12 [97,101]. The lymphocyte response includes the activation of both CD4 and CD8 lymphocytes [65], and the main cytokine effector is γ-interferon [75], which stimulates the generation of reactive oxygen species (ROS) and nitric oxide (NO) [17]. In some tissues (see below), the cellular response is the formation of a well-structured granuloma with a fibrinoid ring and a central lipid vacuole, with or without polymorphonuclear leukocytes surrounded by a palisade of mononuclear cells with few bacterial structures inside [76].

#### 3.4.3. Chronic Q Fever

From an immunological point of view, chronic Q fever is marked by elevated inhibitory cytokines, mainly IL-10 [19,21,75,76,100]. The cells responsible for IL-10 production are Th2 lymphocytes [102] and regulatory T lymphocytes (CD4+ CD25+ Foxp3) [19,81,103]. Other immunological changes described in patients with chronic Q fever include increased levels of TNFα [21,101] (with an unclear pathogenic role) [19,21], altered granuloma formation [21,63], increased matrix metalloprotease gene expression [21], and potential associations with innate immune system polymorphisms (e.g., TLR1, NOD2 and MYD88) [104]. In addition, other alterations (increased IFN-γ/IL-2 ratios > 11 or B cell marker sCD23 alterations) have been observed, although their significance is unclear [19,21].

People most at risk for chronic Q fever fall into the following three groups: the immunocompromised (although rarely in HIV-infected patients [105]), pregnant women, and patients with endothelial lesions (valvular heart disease or vascular grafts). In the first two cases, modulation of the immune response offers a good explanation for this development. The connection between endothelial involvement and the development of chronic Q fever is more complex. One interesting mechanism postulated is that endothelial injury increases the apoptosis of circulating cells (neutrophils, platelets, and monocytes), which, when phagocytosed by macrophages, triggers an increase in proinflammatory cytokines [106].

#### 3.4.4. Q Fever Fatigue Syndrome (QFS)

The pathogenesis of QFS is not fully understood, partly because there are no unambiguous criteria for defining this syndrome, and objective data are scarce and sometimes contradictory [107,108,109,110]. Various alterations in the immune system have been implicated, including the following: (*i*) the presence of non-viable genetic and antigenic *C. burnetii* material; (*ii*) the increased production of several cytokines, mainly IL-6 and, to a lesser extent, TNFa and IL-1b; (*iii*) the association with genetic factors, such as a higher frequency of HLA-DRB1*11; and (*iv*) the detection of markers indicating mitochondrial dysfunction (e.g., decreased humanin).

## 4. Pathophysiology

This section reviews the mechanisms of injury, as well as the clinical and biological manifestations of both the acute and chronic forms of Q fever, with a brief mention of the associated Q fever fatigue syndrome.

### 4.1. Mechanisms of Injury (Figure 9)

#### 4.1.1. Direct Cell Damage

As explained above, the replication of *C. burnetii* within alveolar macrophages eventually leads to their destruction. However, there is no clear evidence of direct cytoxicity in other infected cell types (such as hepatocytes).

**Figure 9:**
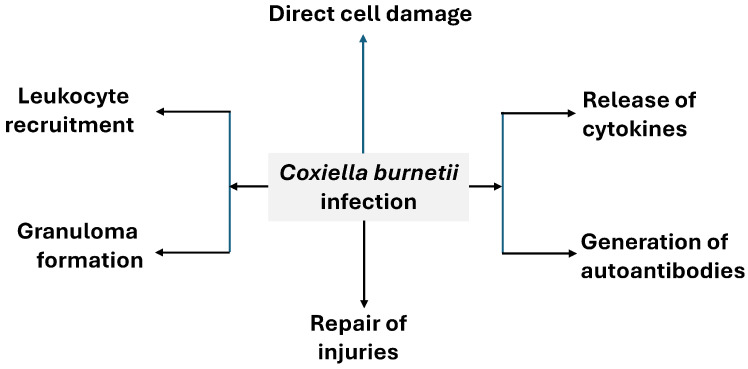
General pathophysiology of Q fever.

#### 4.1.2. Leukocyte Recruitment [78]

In Q fever patients, inflammatory cell infiltration has been described in different tissues (lung, endocardium). In acute forms, the most common findings in the lung are (*i*) the epithelial hypertrophy of the bronchioles, (*ii*) the presence of edema, (*iii*) bronchiolization of the alveolar epithelium, and (*iv*) the appearance of inflammatory infiltrates, characterized by the presence of round cells (lymphocytes, macrophages) and plasma cells [111,112]. Chronic Q fever with endocarditis is often marked by local mononuclear cell infiltrates [113].

#### 4.1.3. Granuloma Formation

Granulomas are organized accumulations of macrophages that have differentiated into epithelioid cells and can be accompanied by other inflammatory cells and associated phenomena (such as giant cell development and necrosis) [114]. The presence of granulomas in Q fever is characteristic of hepatic, cutaneous, and bone marrow involvement, and their most characteristic, but not pathognomonic, form is the “doughnut” [115,116,117,118], a central lipid vacuole, with or without polymor-phonuclear leukocytes, surrounded by a palisade of mononuclear cells. However, in pulmonary or endocardial involvement, the presence of granulomas is exceptional. The formation of well-structured granulomas is associated with the rapid resolution of infection.

#### 4.1.4. Cytokine Release

The production of proinflammatory cytokines by mononuclear cells, especially IL-6 and TNFa, is associated with common symptoms of acute inflammation, such as fever, fatigue, poor general condition, and myalgias [97]. It has also been observed that, in acute forms of Q fever with hepatic involvement, cytokine production is higher than in forms with isolated fever or pneumonia [21,101]. From a clinical point of view, this finding supports the use of a course of prednisolone if apyrexia is not achieved after three days of antibiotic therapy [100]. Acute Q fever is characterized by a distinctive inflammatory marker pattern, with significantly increased serum CRP (C-reactive protein) levels, but serum procalcitonin levels and white blood cell count are in the normal range [119,120].

Another interesting aspect is the association between elevated IL-10 production in chronic forms with the development of lymphoma, especially diffuse large cell lymphoma, although the data on this association are not entirely conclusive [2,121].

#### 4.1.5. Generation of Autoantibodies

In a significant number of patients, the stimulation of B lymphocytes not only leads to the production of specific antibodies, but it is also associated with the generation of autoantibodies [100]. Antibodies against mitochondrial antibodies, cold agglutinins and antiphospholipid antibodies, ANA (antinuclear antibodies), and rheumatoid factor have been described [2,122,123]. In acute Q fever, these autoantibodies have been associated with rheumatologic manifestations and thrombotic phenomena, and in chronic Q fever, they have been associated with the development of endocarditis.

#### 4.1.6. Repair of Injuries

In chronic Q fever, the repair of damaged tissues manifests as the development of valvular fibrosis, often with calcifications and small-sized vegetations [113].

### 4.2. Clinical and Biological Manifestations

#### 4.2.1. Acute Q Fever

The diagnosis of acute Q fever is based on the combination of the objective evidence of recent infection and a compatible clinical picture.

Proof of recent infection is usually provided by serological techniques (mainly indirect immunofluorescence) that demonstrate antibody seroconversion (a 4-fold increase in antibody titers) or elevated IgM titers against *C. burnetti* phase II antigens [119]. However, serological techniques can provide both false-negative and false-negative results. The main cause of false negatives is the long period (1–4 weeks) between the onset of symptoms and antibody detection [76,119,124,125,126]. In this context, the detection of *C. burnetii* genetic material, mainly by PCR, is a valuable tool for the early diagnosis of acute Q fever [124,127,128]. On the other hand, cross-reactivity between *C. burnetii* with other bacterial genera such as *Legionella, Rickettsia*, or *Bartonella* has been described, which may lead to false-positive results [129,130,131]. Certainly, a previous infection with *C. burnetii* could be a reason for a false-positive result.

The incubation period for acute Q fever is from two to four weeks [18,132]. Clinical manifestations are more common in middle-aged males than females and in the extremely young and old (children and the elderly) [1,2,133]. The three main clinical forms of acute Q fever are nonspecific febrile illness, pneumonia, and hepatitis [1,2,15,76,134] (Table 2).

Nonspecific febrile illness is a self-limited, flu-like syndrome with no evidence of pneumonia or hepatitis. The most frequent symptoms are fever, headache, malaise, fatigue, and myalgia [2,21,132]. The production of proinflammatory cytokines is the main mechanism behind these manifestations. Fever is the most common symptom and is high (often up to 40 °C), with a variable duration (between 5 and 57 days, with a median of 10, higher with increasing age). Headache is usually retro-orbital and sometimes very intense (described as the most severe pain ever experienced). Pneumonia is a common form in some geographical areas. The diagnosis of acute Q fever pneumonia requires clinical data suggesting pulmonary involvement and alterations on chest radiology, usually associated with the systemic manifestations described previously [2,132,135,136,137,138]. The recruitment of inflammatory cells is the primary mechanism involved, probably with the local effects of pro-inflammatory cytokines (Table 2). The spectrum of acute Q fever pneumonia ranges from very mild (nonproductive cough, fever, and few auscultatory abnormalities) to very severe (acute respiratory distress) [132,135]. Radiological findings are highly polymorphic. A key feature is the presence of distinctive lobar or segmental alveolar opacities (often oval in shape, known as “round pneumonia”) with a preference for the lower lobe. These data are useful in diagnosis to differentiate it from other atypical pneumonias (with an interstitial pattern and often bilateral distribution) [132,135,136]. An association with pleural effusion is not unusual, but it varies significantly depending on the published series [2,135,136,137]. In the ancillary studies, some data are more diagnostic, such as normal or low leukocyte counts and elevated C-reactive protein (CRP) levels [2,135]. Other analytical data described in acute Q fever pneumonia include altered transaminases (around 20% of pneumonia cases), hyponatremia (probably related to the inappropriate secretion of antidiuretic hormone) [135] and elevated serum ADA (adenosine deaminase) levels [138]. Q fever hepatitis is not well defined. Elevated transaminase levels (twice the upper limit of normal or higher) are the criterion most commonly used for diagnosis [1,2,15,139,140]. The mechanisms involved include granuloma formation, the recruitment of inflammatory cells, and the production of inflammatory cytokines (Table 2). Hepatomegaly (painless or painful) or jaundice are highly variable clinical signs depending on the series [1,2,15,139,140]. Apart from hypertransaminasemia, other analytical data reported are elevated serum bilirubin and, above all, elevated alkaline phosphatase activity (dissociated cholestasis) [15,137].

Other less common clinical forms (<1% of cases) of acute Q fever are shown in Table 2. In most cases, the underlying mechanisms are speculative. The main manifestations include cardiac (pericarditis, myocarditis) [141,142], neurological (meningitis, meningoencephalitis, Miller Fisher syndrome, myelitis, cranial or peripheral neuritis, Guillain–Barré syndrome) [143,144,145], ocular (uveitis) [145], dermatological (rash, erythema nodosum, nodular panniculitis) [2,76,146,147,148], hematological (hemolytic anemia, hemophagocytic syndrome, hypoplastic anemia, bone marrow necrosis, acute lymphadenitis) [149,150,151,152,153], nephrological (acute glomerulonephritis, interstitial nephritis) [149,154], splenic (abscess, infarction, rupture) [155,156,157,158], gallbladder [159], or rheumatological (rhabdomyolysis, polyarthritis, vasculitis) [122,159,160,161,162,163].

#### 4.2.2. Chronic Q Fever

There is no clear definition of chronic Q fever, probably due to the limited number of cases in published series and the diagnostic criteria used [164,165,166,167]. A standard reference is the time frame, considering a six-month interval from the onset of an acute form as a temporal criterion, although the absence of this antecedent is not uncommon [132,165]. Given these nuances, the incidence of chronic Q fever is between 1 and 5% of cases of acute Q fever [21,132,164,167].

The two main clinical forms of chronic Q fever are endocarditis and vascular infection. Although both osteoarticular involvement and immunosuppression are well documented, they occur very rarely [168,169,170]. The role of pregnancy is unclear [171,172,173], and chronic liver disease is not considered a form of chronic Q fever [164,168].

The diagnostic criteria for both main forms involve demonstrating active *C. burnetii* infection (by PCR and/or serology) and the presence of endocardial/vascular involvement (by echocardiography or other imaging techniques) [164,165]. Both criteria must be met simultaneously for a definitive diagnosis. However, there are several limitations in application. The PCR detection of genetic material in tissues is a valuable diagnostic tool, but obtaining samples often requires invasive techniques, which can be a limitation. The use of this technique in peripheral blood is more questionable because it can lead to false positives (due to contamination) [165] and false negatives if serum is used instead of anticoagulated blood [124]. Although it has been previously mentioned in the diagnosis of acute Q fever, one cause of false-negative PCR results is the absence of bacteremia at the time of evaluation. For serological diagnosis, the most commonly used criterion is the presence of elevated titers of antibodies to phase I antigens; indeed, the presence of titers > 1:800 is one of the major Duke criteria for the diagnosis of infectious endocarditis [174]. However, several factors should be borne in mind when making a determination. First, the reliability of the results depend on the technique (IFA is recommended) and the method (in-house versus commercial, and within the commercial methods, the specific reference laboratory) used [167,175]. Second, the cut-off titer is variable; values < 1:800 do not exclude the diagnosis of chronic Q fever, values ≥ 1:1024 are more sensitive but less specific, and values > 1:6400 are more specific but less sensitive [167]. In terms of imaging studies, echocardiography is a useful tool for evaluating heart conditions, although with some limitations. Transthoracic echocardiography (TTE) is a non-invasive, bloodless test that is widely used. The main disadvantage is that the vegetations detected are small in size, and they are visualized in fewer than one third of patients [132,165,168]. Some authors suggest that echocardiography screening no longer be undertaken in patients with acute Q fever [176]. Nevertheless, transthoracic echocardiography can detect previously unknown valve lesions (associated with rheumatic heart disease, bicuspid aortic, and mitral valve prolapse) [165,177,178]. Transesophageal echocardiography (TEE) is an invasive technique that should be used when other imaging modalities are inconclusive. Although there is no established consensus, the following criteria have been used for performing TEE: (*i*) age (older than 40–45 years) [165,179], (*ii*) antibody titer development (3–6 months) [179,180], and (*iii*) Positive IgG aCL (anticardiolipin antibodies) [179]. Other useful imaging tests are CT (computed tomography), MRI (magnetic resonance imaging), and especially PET (positron emission tomography) for vascular lesions [164,181,182]. The need to combine microbiological data with clinical manifestations to make a definitive diagnosis is emphasized [165,183].

The usefulness of other techniques (i.e., IgA antibody detection after interferon stimulation by ELIspot) in the diagnosis of suspected chronic Q fever has also been described [184,185], although none are currently considered useful in clinical practice [186].

The main manifestation of chronic Q fever is infective endocarditis. The clinical features are markedly different from other forms of this syndrome, with left ventricular failure in about half of patients, arterial embolism in 20% of patients, as well as fever (often low-grade and intermittent) and evidence of systemic inflammation (anemia, thrombocytopenia, polyclonal hyperglobulinemia, and an elevated erythrocyte sedimentation rate in almost all cases) [168,177]. Hepatic transaminases and alkaline phosphatase are also usually elevated.

#### 4.2.3. Q Fever Fatigue Syndrome

QFS involves persistent fatigue that develops after the acute Q fever infection has cleared. It is characterized by the presence of subjective manifestations but no objective focus of infection or current evidence of *Coxiella burnetii* infection [187,188]. The pathophysiology of this syndrome is poorly understood.

## 5. Conclusions

The original name of human infection with *Coxiella burnetii* was Q (query) fever to denote the unknown aspects of this infection. This name is still used today. In recent decades, many of these unknown aspects have been clarified, which we have summarized in this article. In addition to acknowledging the remaining unknowns in Q fever, it would be valuable to explicitly highlight the current knowledge gaps and the research underway to address them. Key unresolved issues include understanding why the clinical manifestations of acute *Coxiella burnetii* infection range so widely, from asymptomatic cases to mild/moderate illness and severe complications. It also remains unclear whether clinical outcomes differ depending on the duration between symptom onset and etiological diagnosis, and it is also unknown whether age plays a significant role in the presentation of acute Q fever. Another critical area of research concerns the disproportionately higher incidence of chronic Q fever in pregnant women, which raises the question of whether systematic screening should be considered during pregnancy in endemic areas. Moreover, there is an urgent need to reach a consensus on the definition of Q fever fatigue syndrome and to identify reliable biological markers for its diagnosis and monitoring. In the case of chronic Q fever, standardized recommendations for microbiological and imaging studies are still lacking, as is guidance on prophylactic or therapeutic interventions based on their results. Ongoing and future studies by various scientific groups aim to address these questions, which will be crucial for improving diagnostic precision, clinical management, and ultimately reducing the disease burden of Q fever. As research continues, it is likely that many more aspects will be clarified in the coming years.

## Figures and Tables

**Table 1:** *Coxiella burnetii* variants.

	SCV (*Small Cell Variants*)	LCV (*Long Cell Variants*)
	** 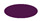 **	** 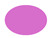 **
**Morphology**		
Phenotype	Mainly Smooth	Mainly Rough
Size	0.2–0.5 µm	>1 µm
Shape	Bacillar	Pleomorphic
**Structure**		
Outer membrane proteins (Omp)	Omp34	OmpA
Internal proteins	Hq1 and ScvA	EF-Ts and EF-Tu
Chromatin	Condensed	Dispersed
**Functional aspects**		
Metabolic activity	Low	High
Environmental stability	High	Low
**Immune response**		
Activation of the complement system	No	Yes
TLR 2 binding	No	Yes
Overall effect	Mainly Immune evasion	Mainly Immune activation

EF-Ts: Elongation factor thermo stable; EF-Tu: Elongation factor thermo unstable; Hq1: Histone-like protein q1; ScvA: Small cell variant protein A; TLR 2: Toll-like receptor type 2.

**Table 2:** Clinical manifestations of acute Q fever.

Frequency	Clinical Manifestations		Reference/s
**>1%**	**Nonspecific febrile illness**		[2,21,132]
	**Pneumonia**		[2,132,135,136,137,138]
	**Hepatitis**		[1,2,15,139,140]
**<1%**	**Cardiac**	Pericarditis	[141]
		Myocarditis	[142]
	**Neurologic**	Meningitis/Meningoencephalitis	[143]
		Miller Fisher syndrome	[144]
		Cranial or peripheral neuropathy	[143,145]
		Guillain Barre syndrome	[143]
	**Ocular**	Uveitis	[145]
	**Dermatologic**	Rash (maculopapular or vesicular)	[2,76]
		*Erythema nodosum*	[146,147]
		Lobular panniculitis	[148]
	**Haematologic**	Haemolytic anemia	[149]
		Hemophagocytic syndrome	[150]
		Hypoplastic anemia	[151]
		Bone marrow necrosis	[152]
		Acute lymphadenitis	[153]
	**Nephrologic**	Acute glomerulonephritis	[154]
		Interstitial nephritis	[149]
	**Abdominal**	Splenic abscess	[155]
		Splenic infarction	[156]
		Splenic rupture	[157,158]
		Acalculous cholecystitis	[159,160]
	**Rheumatologic**	Rhabdomyolysis	[161,162]
		Polyarthritis	[163]
		Vasculitis	[122]

## Data Availability

No new data were generated or analyzed in support of this review.
